# Clinical efficacy of Platelet-Rich Plasma versus local Methylprednisolone Injection in Lateral Epicondylitis

**DOI:** 10.12669/pjms.39.5.6521

**Published:** 2023

**Authors:** Saeed A. Shaikh, Muhammad Tahir, Nadeem Ahmed

**Affiliations:** 1 Dr. Saeed A Shaikh, FCPS. Department of Orthopaedics, Surgical Building, Jinnah Postgraduate Medical Centre, Rafiqui Shaheed Road, Karachi, Pakistan; 2 Mr. Muhammad Tahir, MRCSEng FCPS. Department of Orthopaedics, Surgical Building, Jinnah Postgraduate Medical Centre, Rafiqui Shaheed Road, Karachi, Pakistan; 3 Dr. Nadeem Ahmed, FCPS. Department of Orthopaedics, Surgical Building, Jinnah Postgraduate Medical Centre, Rafiqui Shaheed Road, Karachi, Pakistan

**Keywords:** Lateral epicondylitis, Tennis elbow, Pain, PRP, Steroids, NRPS, qDASH

## Abstract

**Objective:**

To compare the results of local administration of platelet-rich plasma (PRP) with methylprednisolone in the treatment of tennis elbow.

**Methods:**

This retrospective cohort was conducted at Jinnah Postgraduate Medical Center (JPMC) during January 2017 to April 2018. Patients conservatively managed for lateral epicondylitis with local methylprednisolone injection or PRP injection were approached for possible inclusion in the study at 12 months of treatment. The primary outcome of the study was to determine the Numerical Pain Rating Score (NPRS) on resisted wrist extension. Whereas, the secondary outcomes were quick disability arm, shoulder, and hand score (qDASH), the grip strength and VAS for satisfaction. The baseline, six weeks and three month data on Grip strength, NPRS, and qDASH were extracted from the patients’ medical records maintained at the hospital. The data were analyzed by using SPSS software.

**Results:**

A total of 91 patients were approached, of them 81 (89.01%) agreed to participate. There were 46 (56.79%) who received local methylprednisolone injection and 35 (43.20%) received PRP. At 12 months follow up, there was no difference in NPRS pain scores between the two groups (p=0.691); pain decreased in both groups at six weeks and at 12 months. There was no significant difference in the functional outcome (qDASH score) in both groups. Both groups were equally satisfied with the treatment they had received.

**Conclusion:**

The study concluded that there is no difference between outcome and efficacy of both treatment modalities used for the treatment of tennis elbow in alleviating pain at 12 months.

## INTRODUCTION

Tennis elbow is usually work-related or sported related pain around the lateral epicondyle that occurs due to local injury, overuse, hypervascularity and gripping activities of the wrist and fingers.^1^,^2^ The treatment options range from conservative management to surgical therapy. However, there is no consensus that single treatment modality is superior to the other in effectively treating lateral epicondylitis.^3^,^4^

Steroids tend to provide immediate relief of symptoms; however, their effect is short term and lacks a long-term benefit.^5^ On the other hand, autologous platelet-rich plasma has shown promising results in various human trials. It has been applied to various tissues such as tendon injury, muscle strain, osteoarthritis, bone healing.^3^,^4^ Still, compelling long-term evidence is not available in terms of the efficacy on which method is the preferred treatment for lateral epicondylitis.^6^-^8^ Therefore, the study aims to investigate the efficacy of PRP with local steroid injection.

## METHODS

This retrospective cross-sectional study was conducted at Jinnah Postgraduate Medical Center (JPMC) between January 2017 and April 2018. Patients who were previously treated for tennis elbow in outpatient departments (OPDs) were contacted for a one-year follow-up to measure patient-reported outcomes and to evaluate the clinical efficacy of the two treatments.

### Ethical Approval:

The study was approved by the institutional review board of Jinnah Postgraduate Medical Centre, Karachi with reference number (No. F.2-81/2022-GENL/166/JPMC). An informed consent was obtained from all participants.

Patients aged 18 years and above who were treated for lateral epicondylitis were contacted and eighty-one patients gave their informed consent to be followed up at one-year by applying convenience sampling technique.

### Inclusion and Exclusion Criteria:

Patients diagnosed with tennis elbow having active complaints for more than six months or patients that had exhausted other options like physiotherapy, orthosis, taping and lifestyle modification, NSAIDs or pain refractory after avoiding any manual work for six to give appropriate rest were included in the study. Patients that did not consent for their data to be included or the ones that did not agree for a one-year follow-up were excluded from the study.

For the PRP group (n=35), 20 milliliters of venous blood collected from the cephalic vein under aseptic measures using a scalp vein catheter as it avoided turbulence when collecting blood and the blood was collected into two citrate tubes having 0.9% sodium citrate as anticoagulant. was then centrifuged till plasma concentrates were obtained. The surgeon followed the following protocol as illustrated by Dhurat and Sukesh^9^:

The collected blood was spun twice, the first time at 1000 rpm for 15 minutes to separate the red blood cells from the other components. After the first spin, the total blood separated into: an upper layer of platelets and WBCs, an intermediate buffy coat layer of WBCs, and a bottom layer of RBCs. An empty sterile tube was used to make pure-PRP (P-PRP). The buffy coat and a few RBCs were transplanted to make leucocyte rich (L-PRP). Step two is the strong spin step, 2000 RPM for five minutes. The tube’s spin should be just enough to help create soft pellets (erythrocyte-platelet). The volume containing predominantly PPP (platelet-poor plasma) was eliminated. PRP is made by homogenising pellets in lower 1/3 (5 ml plasma) (Platelet-Rich Plasma). The was the surgeon’s preferred way of producing autologous PRPs.

For the steroid group of patients (n=46), methylprednisolone 80mg along with 3cc of 2% lignocaine was injected. After aseptic measures, the surgeon injected PRP or corticosteroid into the insertion of the affected ECRB tendon with a 22G needle.

As a standard practice of the treating surgeon, baseline collection of the numerical pain rating score (NPRS) at resisted wrist extension, grip strength and quick disability arm, shoulder, and hand score (qDASH) were collected at the first visit before offering any treatment, and at six weeks and three months interval after treatment. Only on episode of injection was given to the patient in order to keep the study simple for the inference of the results and avoid the confounding effects of subsequent injections.

Tennis elbow is diagnosed using patient history and clinical findings, according to National Institute for Health and Care Excellence (NICE-UK)^10^ November 2020 recommendations. Thus, patients with an insidious onset without any history of gross trauma but due to excessive overuse or increased activity with pain or a burning sensation radiating from the lateral epicondyle to the dorsal forearm worsened by repetitive wrist motions involving gripping activities like opening a bottle cap or jar lid or turning a doorknob. A weak grip for example, difficulty lifting a cup, or lifting bags with an extended elbow, and a history of elbow discomfort and treatments or disrupted sleep may also be symptoms. Common examination findings include tenderness above and/or distal to the lateral epicondyle and along the common extensor tendon. Resisted middle finger extension may reproduce the symptoms. Dorsiflexion of the wrist against resistance while flexing the elbow at 90 degrees - elbow extension would exacerbate pain. Reduced grip strength. However, there would be normal active and passive movement in the elbow and wrist joints.

However, to compare the clinical effectiveness of the treatments the patients who gave consent had their NPRS rated at resisted wrist extension, grip strength and qDASH were collected along with the addition of SF-12 scale at one-year follow-up.

The main outcome of the study was NPRS which is the most used and easy-to-administer instrument to quantify pain of an individual suffering from any illness. The ranges from 0 to 10, with zero being “no pain” and 10 being “the worst pain imaginable. NPRS has a verbal version and a written version.^11^ The surgeon’s preferred choice was the verbal version.

The secondary outcomes were quick disability arm, shoulder, and hand score (qDASH) having a range of 0-100 with a higher score denoting a worse outcome^12^, and the grip strength in percentage to the normal contralateral side, the measure by a dynamometer. Visual Analogue Scale for satisfaction is an objective instrument to quantify a person’s satisfaction for a particular treatment option. The score ranges from 0-10 points, with 10 being the completely satisfied with treatment.^13^

Data analyses were performed on SPSS version 22.1 (IBM, Armonk, USA). The categorical data were reported as frequencies and percentages, whereas the continuous variables were provided as mean and standard deviation. A p-value of 0.05 established level of significance while keeping the confidence interval of the study at 95%. The Independent t-test of continuous variables were performed.

## RESULTS

A total of 91 patients were approached to participate in the study, of them 81 patients agreed.. The mean age of participants was 41.17±15.43 years. Forty-six (56.79%) patients received local steroid injections whereas 43.20% (n= 35/81) patients were managed with autologous PRP injection. Females (n=64/81, 79.01%) were more affected and patients with the dominant side limb (n=56/81, 69.1%) presented more to the clinic for the treatment with a mean duration of symptoms 28.86±4.85.

The baseline NRPS on resisted wrist extension (p-value= 0.13) for the steroid group was 4.69 ±1.9 and for the PRP group 4.22 ±1.8, whereas the baseline qDASH (p-value= 0.09) for the steroid group was 43.56±10.84 and for the PRP group was 41.08 ±8.97, likewise the baseline grip strength (p-value=0.70) for the steroid group was 79.13±9.09 and for the PRP group was 78.14±13.87.

There was no difference between the two-treatment arm for NRPS at 12 months; it was also worth noting that the pain decreased throughout six weeks to 52 weeks period in both the groups. Furthermore, participants in the steroid group had significant pain relief as compared to PRP patients at six weeks (p-value 0.012) and 12 weeks (p-value 0.013) interval.

Likewise, there was no significant difference in the functional outcome (qDASH score) between the two groups and the grip strength during the entire follow up period. However, a similar pattern was observed for qDASH, and grip strength as was observed for NRPS at the end of 12 months, The outcomes of the patients is summarized in Table-I.

**Table-I:** Comparison of Patient Reported Outcomes.

Outcomes	PRP (n=35)	Steroids (n=46)	P-value
***Primary outcome***			
NRPS score at 6 weeks	1.95±0.87	1.70±0.39	0.08
NRPS score at 12 weeks	2.69±0.33	2.85±0.59	0.15
NRPS score at 12 months	3.14±0.44	3.09±0.75	0.70
***Secondary outcomes***			
qDASH at 6 weeks	40.75±18.37	39.27±19.28	0.72
qDASH at 12 weeks	34.72±9.85	35.11±13.74	0.88
qDASH at 12 months	28.69±9.50	30.56±8.59	0.35
Grip strength at 6 weeks	82.77±6.84	82.93±9.88	0.93
Grip strength at 12 weeks	81.42±9.68	82.43±9.89	0.64
Grip strength at 12 months	79.34±13.02	81.69±10.16	0.36
VAS for Satisfaction	7.75±1.07	7.70±1.13	0.83

NRPS: Numerical Pain Rating Score, qDASH: Quick Disability Arm, Shoulder and Hand Score, VAS: Visual Analogue Scale for Patient Satisfaction.

**Table-II:** Comparison of Patient Reported Outcomes as per Demographics

Outcomes	Male	Female	P-value	Side 1	Side 2	P-value
	
	N= 17	N= 64		N= 25	N= 56	
***Primary outcome***						
NRPS score at 6 weeks	18.2±4.1	18.1±7.1	0.93	17.0±4.2	18.6±7.3	0.32
NRPS score at 12 weeks	28.0±3.9	27.8±5.3	0.91	26.2±2.3	28.6±5.7	0.044
NRPS score at 12 months	30.3±5.3	31.4±6.7	0.51	31.8±7.8	30.9±5.7	0.57
***Secondary outcomes***						
qDASH at 6 weeks	42.6±17.6	39.2±19.2	0.51	38.8±20.0	40.4±18.4	0.72
qDASH at 12 weeks	35.9±10.2	34.7±12.7	0.71	35.4±13.2	34.7±11.8	0.83
qDASH at 12 months	28.5±7.7	30.1±9.3	0.51	29.6±11.2	29.8±7.9	0.93
Grip strength at 6 weeks	83.3±10.8	82.8±8.1	0.82	83.9±6.4	82.4±9.5	0.47
Grip strength at 12 weeks	83.8±11.1	81.5±9.4	0.40	83.0±7.7	81.5±10.6	0.52
Grip strength at 12 months	79.1±11.8	81.1±11.4	0.53	80.6±11.8	80.7±11.4	0.97
VAS for Satisfaction	76.6±10.8	77.4±11.2	0.79	77.0±9.7	77.3±11.7	0.90

NRPS: Numerical Pain Rating Score, qDASH: Quick Disability Arm, Shoulder and Hand Score, VAS: Visual Analogue Scale for Patient Satisfaction.

## DISCUSSION

The results of this study states that there is no difference between the outcomes of the two treatments i.e., PRP methylprednisolone injection in the management of lateral epicondylitis at 12 months. Lateral epicondylitis is a common condition has a various treatment options ranging from bracing, physiotherapy, local injections to surgery. The findings of the study have a significant implication and potential to inform treating surgeon while choosing the cost-effective treatment option.

Local corticosteroid administration was once considered a gold standard has now become a least considerable option due to its high relapse rate and recurrence of symptoms.^14^ However, numerous trials and meta-analyses have supported the role of autologous PRP as an attractive alternative to local steroid injections. However, there is no conclusive evidence that PRP is a better treatment option than the corticosteroids in the management of tennis elbow.^14^-^18^ Therefore, the study provided evidence to some extent by measuring the outcome of the treatment at 12-month interval. The study was able to compare the pain score and functional outcome score with baseline data help in further decision making and rigor of the study.

The main point of interest was the improvement in pain and subsequent recovery in the functional outcome, which are the most common concerns of the patient. There are few high-level evidence studies which compare the effectiveness of PRP with steroids and supports in favour of PRP injection for lateral epicondylitis^6^,^8^,^15^-^17^. Mishra et al compared PRP with a control group and found promising results favoring which leukocyte-enriched PRP compared with an active control group at 24 weeks.^16^ Peerbooms et al. in another double-blinded randomized controlled trial reported that PRP significantly reduced the pain and improved function in comparison to steroids at 1-year follow-up. The results of Peerbooms et al. are contradictory to the findings of our study.^8^

However, a recent randomized controlled trial conducted by Linnanmaki et al. in 2020; compared the effect of PRP, autologous blood and saline injections (placebo) in the treatment of lateral epicondylitis.^18^ The trial found no clinically important in VAS pain scores and functional outcomes of 119 patients when compared with the placebo group at 52 weeks thus recommending against the use of PRP or autologous blood for the treatment of lateral epicondylitis. The results of Linnanmaki et al. also hold true for our trial.^18^

Another level one evidence study evaluated the effect of PRP, glucocorticoid or saline in reducing pain in patients with lateral epicondylitis.^14^ They concluded that a single injection with either PRP or glucocorticoid was not significantly superior to saline injection for reduction of pain for patients with lateral epicondylitis. The drawback of their study was short follow up of three months due to dropout of large number of patients in all three-treatment arms which again signifies that the treatment given was not able to provide expected pain relief to the patients.

In addition to the NRPS score, our study did not yield any beneficial improvements in the secondary outcomes (functional outcomes) of the trial. These findings are consistent with Linnanmaki et al. and three other studies.^18^-^21^

### Limitations of study:

We did not quantify the pre-centrifuge and post-centrifuge concentration of the PRP. Therefore, we were unable to calculate a standard dose of PRP. Finally, we did not include patients who were refractory to corticosteroids therapy. Hence, we could not know if PRP works in refractory cases of tennis elbow.

### Strengths of study:

Firstly, our study had 89.02% participation at 12 months period. Secondly, this trial used validated patient reported outcomes tools such as NRPS, qDASH and VAS for satisfaction. Finally, and most importantly, the study has a long term follow of 12 months which is plenty of evidence to show the effect of the study.

## CONCLUSION

This trial concludes that platelet-rich plasma is not superior to corticosteroid injection in alleviating pain at 12 months. Findings of this study advocate against the use of orthobiologics in lateral epicondylitis unless a meaningful randomized trial with significant results is published in the future, or a metanalysis with compelling evidence is presented. Till then, we urge the surgeons to opt for evidence-based practice and cost-effective treatment options such as corticosteroids.

### Author`s Contribution:

**SAS:** Recruited patients and did PRP, reviewed the initial and final draft, final approval of the study and is responsible and accountable for the accuracy and integrity of the work.

**MT:** Conception of study, writing, results, interpretation bibliography, final approval of manuscript

**NA:** Supervision of study and administered local steroid injections, validation of study, final approval of the manuscript.
